# Self-identity, lived experiences, and challenges of breast, cervical, and prostate cancer survivorship in Mexico: a qualitative study

**DOI:** 10.1186/s12885-020-07076-w

**Published:** 2020-06-22

**Authors:** Felicia Marie Knaul, Svetlana V. Doubova, María Cecilia Gonzalez Robledo, Alessandra Durstine, Gabriela Sophia Pages, Felicia Casanova, Hector Arreola-Ornelas

**Affiliations:** 1 Sylvester Comprehensive Cancer Center. University of Miami, Miami, FL USA; 2 Department of Public Health Sciences, Leonard M. Miller School of Medicine, Miami, FL USA; 3 Institute for Advanced Study of the Americas, University of Miami, Coral Gables, FL USA; 4 Tómatelo a Pecho & Mexican Health Foundation (FUNSALUD), Mexico City, Mexico; 5 Epidemiology and Health Services Research Unit, CMN Siglo XXI, Mexican Institute of Social Security, Av. Cuauhtemoc 330, Col. Doctores, Del. Cuauhtemoc, 06720 Mexico City, Mexico; 6 Center for Research in Health Systems, National Institute of Public Health, Av. Universidad 655 cerr, Los Pinos y Caminera, Col. Santa María Ahuacatitlán C.P, 62100 Cuernavaca, Morelos Mexico; 7 Catalyst Consulting Group, LLC, 1 Bond Street, PHD, New York, NY USA; 8 Department of Public Health Sciences, Institute for Advanced Study of the Americas, University of Miami, Coral Gables, FL USA; 9 Department of Sociology, University of Miami, Coral Gables, FL USA; 10 Mexican Health Foundation (FUNSALUD), Mexico City, Mexico; 11 Tómatelo a Pecho, Mexico City, Mexico; 12 Centro de Investigación en Ciencias de la Salud, Facultad de Ciencias de la Salud, Universidad Anáhuac Mexico Campus Norte, Naucalpan de Juárez, Mexico

**Keywords:** Cancer, Survivorship, Self-identity, Experiences, Challenges, Mexico

## Abstract

**Background:**

Qualitative research on cancer patients’ survivor-identity and lived experiences in low- and middle-income countries is scarce. Our study aimed at exploring the concept and experience of survivorship for Mexicans living with breast, cervical, and prostate cancer.

**Methods:**

We conducted a qualitative study in Mexico City, Morelos, Nuevo León, and Puebla. The participants were breast, cervical, and prostate cancer patients ≥18 years of age with completed primary cancer treatment. Data were collected via in-depth interviews and analyzed using an inductive thematic approach.

**Results:**

The study included 22 participants with a history of breast, 20 cervical, and 18 prostate cancer. Participants accepted the term “cancer survivor” as a literal interpretation of being alive, medical confirmation of treatment completion, or achievement of a clinical result possibly indicative of cure. The majority of respondents perceived that the future is out of their control and under God’s will. They linked cure to divine intervention and did not demonstrate the sense of empowerment that is often associated with the survivorship term. The principal themes of their narratives encompass: 1) adverse physical and sexual experiences; 2) emotional problems; 3) cancer-related stigma; 4) challenges to obtaining health-related information; 5) financial hardship; and 6) experience of strengthening family ties in order to provide them with support. In addition, women with breast cancer reported distress caused by changes in body image and positive experience with support groups.

**Conclusion:**

In Mexico, cancer patients report complex survivorship experiences that demand post-treatment follow-up and support. There is the need to implement comprehensive, culturally-relevant survivorship programs focused on emotional, informational, and in-kind support and empowerment of cancer patients.

## Background

The incidence of cancer cases continues to rise globally. Simultaneously, improvements in cancer diagnosis and treatment, and in some countries access to that treatment, have increased in the number of people living with cancer [1]. In 2018, there were almost 30 million 3-year and 44 million 5-year cancer survivors worldwide—nearly half of them living in low- and middle-income countries (LMICs) [2].

The growing population of individuals with a history of cancer has raised concerns over post-treatment needs and challenges related to their physical, psychological, and social wellbeing [3–9]. Most research comes from high-income countries and is unlikely to be fully applicable to LMICs as the experiences and challenges of cancer survivorship are culturally constructed and embedded in the existing in social context [10]. Contextually and historically situated circumstances contribute to people’s health beliefs, illness experiences, and behaviors [11, 12] and to the ways in which they adapt to living with a chronic illness such as cancer .

The term “survivor” was proposed by advocacy groups in the United States (U.S.) to encourage empowerment, hope, and solidarity between people with cancer [13–16]. Cancer patients in the U.S. [3, 6, 7, 17], Brazil [18], and Puerto Rico [15], are more prone to adopt the “survivor” identity than those in other settings, such as the United Kingdom [19, 20] and Australia [21, 22]. The proposed “survivor” self-identity concept has been found to be associated with positive mood, satisfaction with life, and self-care [10, 14, 22]. Defining when patients should be deemed “survivors” in the cancer clinical trajectory is highly debated, with some definitions proposing cut-off-points of 3 or 5 years after cancer diagnosis [23], and others considering that patients become “survivors” after completion of primary cancer treatment [24] or even since cancer diagnosis, thus encompassing acute, extended, and permanent stages of survival [13]. Those who reject the term “survivor” typically identify themselves as a cancer patient, a person who is living with cancer, had and no longer has cancer, or a cancer victim [16, 22].

Mexico is an upper-middle income country that shares historical and cultural features with other Latin American (LA) countries, while also possessing unique characteristics. Like many LA countries, Mexico has a history of Spanish colonization characterized by exploitation and domination of indigenous population, which in turn explains the predominance of the Spanish language, Catholic religious identity, and ethnically-mixed population (*mestizos*). Common cultural values and beliefs include an attachment to family (*familismo*) [25] and gender roles that legitimize male dominance and justify female subordination (*machismo* and *marianismo*). Mexico is also quite unique among low and middle income countries in the depth and breadth of health insurance coverage, and particularly cancer through the *Seguro Popular* so that survivorship is becoming an increasingly important issue even among lower income groups [26].

Research from Mexico and other LA countries on cancer survivorship self-identity, lived experiences, and challenges is scarce [14]. One study from Puerto Rico of 23 young adults with a history of cancer treatment found that the most important aspects of their post-treatment trajectory were family, faith, and opportunities to help others [15]. A recent qualitative study of 25 young breast cancer patients in Mexico City identified common survivorship experiences including unmet psychological care and informational needs, being less concerned with fertility, increased family support, narrowed social circles, and barriers to employment [6]. In addition, the research on cancer survivorship experiences of Latinos in the U.S. cannot be generalized to Mexican cancer patients due to differences in social context and health system organization between Mexico and the U.S. and the role of acculturation [6].

Qualitative research on cancer patients’ survivor-identity and lived experiences in LACs can deepen understanding of the survivorship phenomenon and on culturally appropriate strategies to address health and social challenges of this population. In this study, we address the research gap on the experience of survivorship in LA, focusing our analysis on Mexico and on breast (BC), cervical (CC), and prostate cancer (PC) as these are among the most common cancers in the region. The study seeks to explore how Mexicans living with BC, CC or PC experience and make sense of survivorship. We want to learn what meaning they attach to the term “cancer survivor”; and what similarities and differences in these topics exist among patients with these three types of cancer.

## Methods

We conducted a qualitative descriptive study through in-depth interviews from September 2014 to February 2015, in Mexico City, Morelos, Nuevo León, and Puebla. These cites were chosen because they have specialized cancer hospitals and varying socio-economic levels: low (Puebla), median (Morelos), and high (Nuevo Leon and Mexico City) [27]. We used a qualitative descriptive methodology to gain insights regarding the target phenomenon by providing its accurate description without imposing a priori conceptualizations [28].

### Participants

The participants of this study were BC, CC, and PC patients aged 18 years of age or over who had completed primary cancer treatment and received a diagnosis at least 1 year prior to the interview. We used purposeful sampling to identify participants from hospital registries with diverse clinical and socio-demographic characteristics with regards to their age, residence, marital status, level of education, length of time since completing primary cancer treatment, type of health insurance (Social Security for people from the formal labour market and their families, and Seguro Popular health insurance for unemployed and informal sector workers without social security). The number of informants for each cancer type was determined by the principle of data saturation (interviews were conducted until data were repeated or redundant) [29].

### Data collection

Data were collected through in-depth semi-structured interviews guided by a list of predetermined, open-ended questions based on study objectives to ensure consistency across interviews. Examples of open-ended questions include the following: “What do you understand by the term ‘cancer survivor’?”; “Do you consider yourself a cancer survivor?”; “At what moment in time did you feel like a survivor?”; “How do you see your life in the future?”; “How have you been feeling lately?”; “Have you had any health problems after finishing cancer treatment?”; “How do you feel emotionally?” The researcher conducting the interviews maintained a receptive attitude, asking participants to elaborate on their unique experiences and statements. The demographic data was collected during the interviews.

Interviews were conducted at times and locations that were most suitable for participants (e.g., hospital rooms, hospital cafeterias, places of work, or homes) and lasted approximately 1 h. All interviews were audio-recorded and subsequently transcribed. Interviews were conducted by three researchers with doctoral degrees in science and expertise in qualitative methodologies who completed a pre-field training session.

### Data analysis

Data were analyzed through inductive thematic analysis [30]. This process comprised five stages: (1) creation of initial codes through an inductive process; (2) search for themes based on their explicit articulation in the interviews and grouping of smaller codes under common themes; (3) revision of themes; (4) definition and classification of themes; and (5) analysis of the content and meaning of the identified themes. The illustrative interview excerpts are labeled according to the cancer types—BC, CC, and PC —as well as cities of residence-- Mexico City (MexCity), Morelos (Mor), Nuevo León (NL), and Puebla (Pueb) and patient identifier.

Three researchers (MCGR, MAB, and SVD) assessed the transcribed texts separately. Individual decisions on emerging themes and classification of responses were cross-checked to ensure consistency and reliability of the coding. In the cases of discrepancy, the classification of responses into themes was corroborated through discussion and collective agreement. During the analysis, we looked for common and specific themes for each group of participants (those with a history of BC, CC, and PC). Finally, we summarized the topics in the thematic map with the aim of making the results easier to comprehend.

### Ethics

The study was approved by the Ethics Committee of the National Institute of Public Health in Mexico (registry number CI:1157). Researchers also obtained permission to recruit participants from each of the participating hospitals. Prior to each interview, participants received information about the aims and nature of the study and relevant ethical considerations. All invited participants agreed to participate and signed an informed consent form.

## Results

The study included 60 participants: 22 with a history of BC, 20 with CC, and 18 with PC. The median age of participants was 51 years for BC, 50 for CC, and 64 for PC, and the median time since diagnosis was 6, 4, and 2 years, respectively. Half (50%) of BC participants, 35% of CC, and 72% of PC were urban residents. Participants with a history of BC were primarily from the states of Nuevo Leon (36%) and Puebla (32%). Those with a history of PC were primarily from Morelos (33%) and Nuevo Leon (28%). CC participants were equally distributed among the four cities. Level of education was higher among BC patients (all had at least completed secondary education) than CC (nearly half lacked any formal education and almost a third had completed only primary school). Among men with PC, 17% had completed primary school, while the rest had completed high school or higher. Over 55% of participants lived with a spouse or a life partner and had children. All participants had health insurance coverage, the majority of BC and CC through seguro popular and the majority of PC through the social security (Table 1).

**Table 1:** General characteristics of study participants

Characteristics	Breast cancer *n* = 22	Cervical cancer *n* = 20	Prostate cancer *n* = 18
Median age, years (minimum–maximum)	51 (41–69)	50 (33–72)	64.5 (53–77)
Median time since diagnosis, years (minimum–maximum)	6 (2–12)	4 (2–9)	2 (1–5)
Residence, n (%)			
Rural	11 (50)	7 (35.0)	13 (72.2)
Urban	11 (50)	13 (65.0)	5 (37.8)
State, n (%)			
Nuevo Leon	8 (36.4)	5 (25.0)	5 (27.8)
Puebla	7 (31.8)	5 (25.0)	4 (22.2)
Morelos	4 (18.2)	5 (25.0)	6 (33.3)
Mexico City	3 (13.6)	5 (25.0)	3 (16.7)
Schooling, n (%)			
Without formal education	0	9 (45.0)	0
Completed primary school	0	6 (30.0)	3 (16.7)
Completed secondary school	14 (63.6)	4 (20.0)	0
Completed high school,	3 (13.6)	1 (5.0)	13 (72.2)
University	5 (22.7)	0	2 (11.1)
Marital status, n (%)			
Married, or live with a life partner	12 (54.5)	12 (60.0)	13 (72.2)
Divorced or single	9 (40.9)	7 (35.0)	2 (11.1)
Widows	1 (4.5)	1 (5.0)	3 (16.7)
Children			
Without children	3 (13.6)	0	1 (5.6)
1–2	12 (54.5)	7 (35.0)	2 (11.1)
≥3	7 (31.8)	13 (65.0)	15 (83.3)
Type of health insurance			
Seguro popular	18 (81.8)	19 (95.0)	3 (16.7)
Social security	4 (18.2)	1 (5.0)	15 (83.3)

**Fig. 1:**
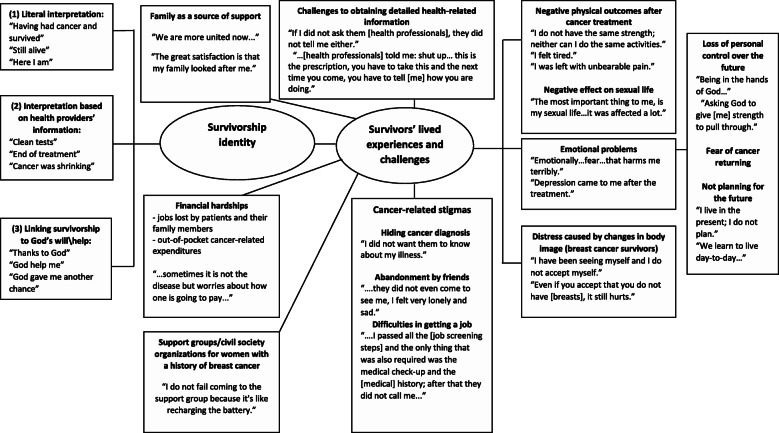
Thematic map of the central topics related to cancer survivorship identity, lived experiences, and challenges in Mexico

Figure 1 summarizes the themes raised by the participants and identified in the thematic analysis. The principal themes of the narratives encompass three types of survivorship identity, six common lived experiences and two themes specific to women with BC.

### Meaning attached to the term “cancer survivor”

One of the central topics of the interview focused on the terminology “cancer survivor” and its relation to survivorship. The term “survivor” did not appear spontaneously in conversation with participants. Although almost all the patients confirmed considering themselves survivors, there were three interpretations of this term: (1) a literal interpretation (“having had cancer and survived”; “still alive”; “here I am”); (2) an interpretation based on health providers’ reporting on their health status (“clean tests”; “end of treatment”; “cancer was shrinking”); and (3) an interpretation, by the majority of participants, linking survivorship to God’s will and support (“thanks to God,”; “God helped me,”; “God gave me another chance,”; “God left me here with a purpose”) (Fig. 1). These interpretations of the “survivor” term were common among participants with different cancer types.

*[Do you consider that you are now a cancer survivor?] “Well, here I am ... as long as it does not appear again it is fine, and if it appears [again] well, it’ll have to be faced...” (PC/Mor-P3).*

*[Do you consider yourself a cancer survivor?] “Well, I would say yes because I already had it and now that I’ve done tests, they came out clean, which means yes.” (PC/MexCity-P4)*

*“... I felt like a survivor when I had surgery; first and foremost, with the hand of God, I am going to pull through...” (BC/NL-P4)*

*[Do you think you are a cancer survivor?] “Yes because God gave me another chance ... from the moment the doctor told me that my tumor was shrinking with the treatment.” (CC/Pueb-P6*).

For some participants, faith in God helped them find meaning in life and cope with the physical and emotional challenges during cancer treatment and recovery.

*[Why do you think you are a survivor?] “Well, I believe that God left me here with a purpose. Maybe he wants me to help a lot of people and perhaps I am here to that end*.” *(CC/NL-P4).*

“*… I know that I have to do my best. God has already put me on this path and there is nothing else but being with Him. I already told God: I am in your hands and make of me what you want. Give me strength to pull through.” (CC/Mor-P4)*

Three participants with PC and one with CC rejected considering themselves as survivors. These participants considered themselves cancer patients; two of them had a low level of education and the other two had a history of metastasis.

### Experiences and challenges of survivorship

The participants described diverse physical, emotional, and social experiences after finishing their primary cancer treatment; most of these experiences were negative.

#### Negative physical outcomes after finishing cancer treatment

Almost half of participants mentioned having adverse physical outcomes after completing their treatment. The most frequent physical problems were lack of strength and pain. The negative physical outcomes were more frequent among BC and CC than among PC participants.

*“... One does not have the same strength nor can one do the same activities as before; one gets tired more ...” (BC/NL-P6).*

“… *everything hurts and I’m just going to tell the doctor because I cannot even sleep.*” (BC/Mor-P4).

In addition, BC, CC and PC participants who were sexually active before cancer diagnosis also reported negative consequences to their sexual life.

*[What has changed in your life since diagnosis that affects your daily activities?] “Well the most important thing for me … is my sexual life. I don’t know, it suddenly took a turn [and] that affected me a lot.” (CC/NL-P4).*

*“My sexual life was over, really, because they cut everything.” (PC/NL-P5)*

Several women with a history of CC, a disease associated with the Human papilloma (HPV) that is sexually transmitted infection, blamed their husbands for their illness:

*“... My husband is the only sexual partner I have ever had, so he transmitted the illness to me. …*” *(CC/ Mty-P4).*

*“... I don’t want to be with my husband again. I’m a housewife. I’m with my children and I take care of him [her husband]. Who knows what women he was been with …*” *(CC/Mty-P2).*

#### Emotional problems

Additionally, several participants mentioned emotional problems, such as fear and distress.

*[How do you feel emotionally?]* “*… it was really a small tumor; they removed it and that’s it. But psychologically...the fear … that hurts terribly ...” (BC/MexCity-P1).*

*"The depression came to me after finishing treatment. It began on the last day of my treatment. …*. [*What did you feel?] “Well, nothing more than pure depression … and tiredness and that’s all.” (PC/MexCity-P1)*

#### Distress caused by changes in body image

A theme specific to BC was distress from changes in body image, as almost half of the participants were struggling to accept themselves after undergoing mastectomy.

*[Has the treatment made you feel different?] “It changes a lot. … I had moments when I cried a lot ... even if you accept that you no longer have them [breasts], it still hurts. The change hurts, it’s a very big change...” (BC/NL-P5).*

*“I had a mastectomy. During the first years, it did not affect me ... I did not care ... [but*] *it’s affecting me now. It’s been a year since I see myself and I do not accept myself. It’s taking a lot of work to accept myself as I am.” (BC/MexCity-P4)*

### Cancer stigmas

We identified several participants who perceived that their relationships with family and friends and opportunities for returning to work were negatively affected by cancer-related stigmas. These outcomes seem to have been at least partly related to an unfounded fear of contagion. This fear could either exist amoung participant communities, or have been perceived by the participants as possibly motivating the behavior of others.

Several participants hid their illness from their friends, expressing worries about being perceived and treated differently. For instance, one participant with breast cancer decided not to tell friends about her cancer because she was afraid they would stop buying the food that she sold. Another participant with prostate cancer was concerned that people would not allow him to enter the public bathroom after finding out about his diagnosis. Others only mentioned that they did not want their friends and coworkers to know.

*“Of my friends and coworkers, there was definitely no one [to support me] because I didn’t want them to know about my illness.” (PC/Mor-P6).*

Another perception of stigma mentioned by some participants was abandonment by friends.

*[Did you have any support from friends and family?] “Not at all... they didn’t even come to see me. I felt very lonely and sad.” (CC/Pueb-P2).*

Several participants who intended to return to work noted barriers, believing that their history of cancer and the possibility of relapse could prevent them from being hired:

*[Will you return to work?] “... I want to return to work. In fact, I am looking for a job. I have already submitted applications and I had some interviews. I had a call from one job and they told me that I passed all the screenings, and that I just needed the medical check and [medical] history; after that, they didn’t call me. I think they didn’t hire me because of my history of cancer.” (BC/MexCity-P3).*

### Financial hardships

Another shared experience was related to the financial hardships caused by patients and family members losing their jobs and by out-of-pocket cancer-related expenditures. Despite most patients having medical costs covered by the seguro popular or social security, the associated, primarily non-medical expenses were a hardship.

Several participants described permanent or temporary job loss, typically without any paid leave. Some who worked prior to their cancer diagnosis described losing their jobs during treatment or had to leave them temporarily. Non-salaried workers described lacking financial support from their employers. Some also discussed loss of income because family members had to give up their jobs to care for them.

Additional causes of financial hardships were out-of-pocket expenses. Participants with Seguro Popular commented on tests and medicines that were temporarily unavailable in hospitals, or analgesics and other symptomatic drugs that were either not covered or only covered for the first 5 years of treatment. Other financial burdens included cost of transportation to treatment and follow-up consultations and housing costs for patients who had to relocate out of their home cities to be closer to hospitals with oncology services:

*“Fortunately, the Seguro Popular paid almost everything, my operation was free; however, we had to pay for several tests and medicines as these were not available.” (BC/Pueb-P4).*

*“When I had radiotherapy, we had to rent a room here in Puebla because [the sessions] were every day for a month and a half, and we couldn’t pay for the transportation. Now I’m still struggling a bit with the transportation [expenses] to come to [medical] consultations.” (CC/Pueb-P5).*

For some patients of low socioeconomic status, these financial hardships triggered feelings of desperation, which led them to borrow or beg for money:

“*… when I came to the emergency room [suffering severe pain], they [health professionals] gave me [analgesics] and they prescribed me Ketorolac but it was not covered through the Seguro Popular insurance. [Did you buy it?] Yes, I bought it, but I struggled to get the money to buy it... I have even begged for money.” (CC/Pueb-P3).*

*“I had [Seguro Popular] insurance for catastrophic expenditures. But they tell me it is only valid for five years and in February it had been five years... it is assumed that after five years you are discharged as a cancer patient, you stop taking medicines and come for follow-ups every six months; but my case is different ... I had to look for [financial] help to get the pills [oral medicines] because they [health insurance] were no longer filling my prescription … ..” (BC/NL-P5).*

### Challenges to obtaining health-related information

The majority of BC and CC, and several PC participants reported difficulties in obtaining health-related information from their providers throughout the treatment process including diagnosis, follow-up and self-care:

“*… If I didn’t ask them [health professionals], they wouldn’t tell me anything.” (BC/NL-P4).*

“*… Sometimes I would ask questions and the doctors would say: ‘I’m going to give you the information you need and don’t ask more … ’”(BC/NL-P3).*

*“Many times they [doctors] would say: ‘shut up, do not talk, do not say anything. This is the prescription; you have to take this, and the next time you come you have to tell me how you are doing’ …*” *(PC/Mor-P2).*

The unmet need for information on how to address physical, emotional, and social challenges prompted many participants to search the internet and join support groups.

### Family as a source of support

The majority of participants spoke positively about their family members as providers of physical, emotional and financial support, and about the challenges they faced as caregivers.

*“She [my daughter] is my nurse, secretary, assistant, maid, everything …*.” *(BC/Pueb-P6).*

“*They [my children] took care of the house … for example, my son is not studying anymore, because he had to [clean] the toilet, wash the bathroom, go shopping …*” *(BC/Pueb-P5).*

Many participants indicated that families became united and stronger after their diagnosis and that this support helped their mood and self-esteem:

*“The family relationship is strengthened.” (CC/Pueb-P5).*

*“We are more united now...” (CC/Pueb-P3).*

*“The great satisfaction is that my family looked after me.” (BC/Pueb-P7).*

*“The support that he gives me as a husband and father of my children, that lifts my self-esteem a lot.” (BC/Pueb-P5).*

### Support groups for women with BC

Several BC patients spontaneously mentioned the importance of support groups, often run by civil society organizations. These groups offered: emotional and informational guidance; training programs related to nutrition, physical activity, and healthy habits; financial support with shelters, transportation, occupational therapy; and, employment opportunities in micro-enterprises.

*“I was going to the psychologist at the “Cruz Rosa” [support group]. This helped me a lot because I was close to committing suicide …*” *(BC/NL-P3).*

*[Has your life changed after the diagnosis and treatment; in what way?] “Yes, for which I’m very grateful because before I did not have time for myself. I used to work and had little time for rest ... and now every month I don’t fail to come to the support group because it’s like recharging my batteries …*” *(BC/Mor-P5).*

Some BC patients joined groups after finishing treatment in order to support other cancer patients:

*“I have come to [the support group] now because I want to help. We mostly provide emotional support.” (BC/Pueb-P2).*

Some CC patients mentioned they failed to find groups for their needs because all the support groups they were for women with BC.

### Loss of control over the future and attitudes toward planning

The majority of participants expressed three closely-related concerns about the future: lack of control over their future, fear of cancer recurrence, and, belief that their future was in the hands of God. They preferred not to plan for the future and instead focused on the present as a coping strategy for uncertainty:

*“... We learn to live day-to-day ... when one lives through this disease, one learns to value and give thanks to God every day, and we no longer make long-term plans, because I cannot say if I will have another month.” (BC/Pueb-P4).*

*“… I live in the present, I no longer make plans for the future …*” *(PC/Mor-P6).*

“*… The only one [who can say how long we’ll live] is up there. He [God] has the last word for us and the life that He gives me, long or short, [will be] welcome.” (CC /Pueb-P3).*

*“God has already put me on this path and there is nothing else but to be with Him. I already told God: I am in your hands and make of me what you want, [and] give me strength to pull through.” (CC/Mor-P4).*

Few participants expressedcontrol over their lives after cancer treatment and plans for the future:

*“Well, we have a lot of opportunities to continue living as long as we do things the way we should … having regular check-ups.” (CC/NL-P5).*

## Discussion

The development and implementation of appropriate health and social strategies to address cancer survivorship in Mexico requires an in-depth understanding of survivor-identity, lived experiences, and challenges.

Our study participants - individuals with a history of breast, cervical, and prostate cancer - generally accepted the term “cancer survivor” as a literal interpretation of being alive or as a medical confirmation of treatment completion and achievement of normal laboratory results. They linked survivorship to God’s help. Their narratives around lived experiences and challenges encompassed several common concerns: 1) adverse physical and sexual experiences; 2) emotional problems; 3) cancer-related stigma; 4) challenges to obtaining health information; 5) financial hardship and job loss; and 6) positive experiences of strengthened family ties and support. Women with BC reported distress caused by changes in body image, as well as positive experiences with support groups.

Embracing cancer survivorship and a “survivor” identity has been cited as important in achieving better physical and emotional outcomes [10, 14, 22]. Although our study participants accepted the terminology, their responses did not reveal the sense of empowerment that is often associated with it [13–16]. The term “cancer survivor” seems more of a label for treatment completion. The majority perceived both their present and the future to be out of their control and under the will of God.

As in studies from other countries [3–9], physical and emotional consequences of cancer and its treatment (e.g. chronic pain, neuropathy, weakness, distress, and depression) were often mentioned by participants. Our participants did not report receiving the continuous, long-term, recommended professional care to prevent and treat these issues [31, 32]. Participants faced serious challenges to obtaining health information.

The results of our study reveal the urgent need for the development and implementation of comprehensive, culturally-relevant survivorship care programs focused on emotional support and empowerment of cancer survivors. Yet, the findings, and an in-depth literature review, show that the Mexican health sector lacks survivorship care programs. Public programming focus primarily on epidemiological surveillance and screening. The National Cancer Institute in Mexico City is the only specialized cancer hospital in the country with a public program for survivorship, and it focuses on BC patients.

The non-governmental organizations with groups are the only other source of informational, emotional, and sometimes financial support for people diagnosed with cancer. Face-to-face and online support groups aim to help cancer survivors increase personal control over the illness and its consequences by sharing illness-related experiences [33]. Previous research found that these groups can help improve coping strategies and reduce psychological distress and depression [34–36]. In our study, only BC patients mentioned participating in and receiving help from support groups, while cervical and prostate cancer patients struggled to find such groups, highlighting the need to develop a focus on different types of cancer.

Our study supports the notion that family support is a key coping strategy to be recognized and encouraged by survivorship programs. In response to a cancer diagnosis, most of the participants’ families mobilized and united to provide support. Previous research has recognized that a positive family response to a stressful situation is usually facilitated by shared beliefs that “make meaning” of the event and foster mutual support and teamwork to achieve recovery [37]. In Mexico, this phenomenon has been documented in families with children with leukemia [38].

Faith in God is another important resources that helps many Mexican cancer patients cope and find meaning in life during treatment and recovery. Studies of Latino U.S. residents also suggest that survivorship interventions should incorporate spirituality as a bridge to resilience [39].

Stigma negatively affects social identity and consequently, psychological wellbeing and achievement of personal goals [40, 41], and our study supports others that recommend dissemination of education as a strategy to reduce the silence and preconceived notions around cancer survivorship [42]. Analogous to findings from other countries, our study found that cancer stigma is “complex and heterogeneous,” affecting different components of patient life [43]. Several participants hid their cancer diagnosis from friends, assuming they would be treated as “contagious”, and then felt abandoned. Several also perceived difficulties getting a job because of assumptions about their productivity after or during treatment [44].

Universal health care should cover comprehensive cancer treatment, includuding survivorship care. Mexico made remarkable progress towards universal health coverage between 2003 and 2018 [26, 45]. All people without social security have the right to health care through the Seguro Popular health insurance and the “Fund for Protection Against Catastrophic Expenditures” (FPCG) also finances cancer care [26, 45]. Our participants stressed the importance of this coverage for their treatment, however they also noted ongoing financial hardships. FPCG covers cancer-specific treatment (e.g., chemotherapy and radiotherapy) 5 years after diagnosis, but does not cover other burdensome expenses, such as symptomatic (e.g., pain-relief) medication, transportation, and housing. In our study, these gaps in coverage disproportionately affected poorer patients, as in commonplace in most of LA because of overreliance on out-of-pocket payment [46]. Recent discusions around closing the Seguro Popular should consider the importance that cancer patients place on this coverage, and not only seek to maintain the program but also expand it to include more aspects of survivorship care.

### Strength and limitations

To ensure the quality of our study in terms of its methodological rigor and transparency we followed Tong et al. COREQ consolidated criteria [47] to develop and report our research. To ensure credibility [48, 49], we used long-lasting engagement of the researchers in the field with participants that allowed building trust and obtaining rich data, and we performed qualitative data analysis through investigators’ triangulation. We described the context so that the study participants’ experiences become meaningful to the readers to allow the transferability of the study results [48, 49]. In addition, the study included participants with diverse socio-demographic and clinical characteristics, and three types of cancers, which allowed for a more comprehensive understanding of a studied phenomenon.

However, our study is limited to BC, CC and PC patients with public health insuarence from four Mexican states; therefore, our findings may not be generalizable to other cancer types, or states, or to those who receive cancer care delivered by private health care providers.

## Conclusion

This study adds to the sparse survivorship literature in Mexico, and the LA region. The results suggest the need to develop and implement comprehensive and culturally consonant survivorship care programs focused on emotional, informational, and in-kind support and the empowerment of cancer patients, especially in the face of stigma. The findings also point to the importance of increasing access to support groups like those that currently operate through civil society organizations. These should not be exclusive to breast cancer patients. Finally, universal health coverage should include comprehensive financial protection for the continnum of care – prevention, early detection, diagnosis, treatment, survivorship and palliative care – that accompanies patients through their cancer journey.

## Data Availability

The intervivwes transcriptions are available upon request.

## Abbreviations

BC: Breast cancer
CC: Cervical cancer
FPCG: Fund for Protection Against Catastrophic Expenditures
LA: Latin American
LMICs: Low- and middle-income countries
MexCity: Mexico City
Mor: Morelos
NL: Nuevo Leon
PC: Prostate cancer
Pueb: Puebla
P: Patient
U.S.: United States
